# Peritoneal tuberculosis with elevated CA-125 mimicking ovarian cancer with carcinomatosis peritonei: Crucial CT findings

**DOI:** 10.17179/excli2016-625

**Published:** 2016-11-16

**Authors:** Jerry Chin-Wei Chien, Chia-Lang Fang, Wing P. Chan

**Affiliations:** 1 Department of Radiology, Wan Fang Hospital, Taipei Medical University, Taipei, Taiwan; 2 Department of Radiology, School of Medicine, College of Medicine, Taipei Medical University, Taipei, Taiwan; 3 Department of Pathology, School of Medicine, College of Medicine, Taipei Medical University, Taipei, Taiwan; 4 Department of Pathology, Wan Fang Hospital, Taipei Medical University, Taipei, Taiwan

**Keywords:** carcinomatosis peritonei, computed tomography (CT), ovarian cancer, peritoneum, tuberculosis

## Abstract

Preoperative diagnosis of peritoneal tuberculosis is often difficult because of confusion with ovarian cancer. A 56-year-old woman was admitted to our hospital with abdominal fullness. Ascites, prominent bilateral ovaries, and elevated CA-125 were noted. Computed tomography showed thickened peritoneum and strandings in the mesentery and omentum. Exploratory laparotomy was performed under the provisional diagnosis of ovarian cancer, but the final diagnosis was peritoneal tuberculosis. Careful evaluation of bilateral fallopian tubes and ovaries and peritoneum are helpful for correct diagnosis.

## Introduction

Diagnosis of extrapulmonary tuberculosis is difficult. Tuberculous peritonitis, accounting for 1 %-2 % of all tuberculosis cases, is caused by abdominal or pelvic tuberculosis that involves the peritoneum (Koc et al., 2006[4]; Oge et al., 2012[8]). The postulated mechanism is tubercular bacilli reaching the peritoneal cavity via the bloodstream or by direct spread from contiguous infected small intestine, lymph nodes, and fallopian tubes (Teh et al., 1987[10]). Patients usually have nonspecific symptoms and signs such as abdominal pain, abdominal distension, and poor appetite. The serum cancer antigen (CA)-125 can be elevated in both ovarian cancer and peritoneal tuberculosis. Similar clinical and image findings lead to diagnostic difficulty and the challenge of distinguishing these two disease entities. We report computed tomography (CT) imaging characteristics of a case of peritoneal tuberculosis with elevation of CA-125 mimicking ovarian cancer with carcinomatosis peritonei.

## Case Report

**Figure 1:**
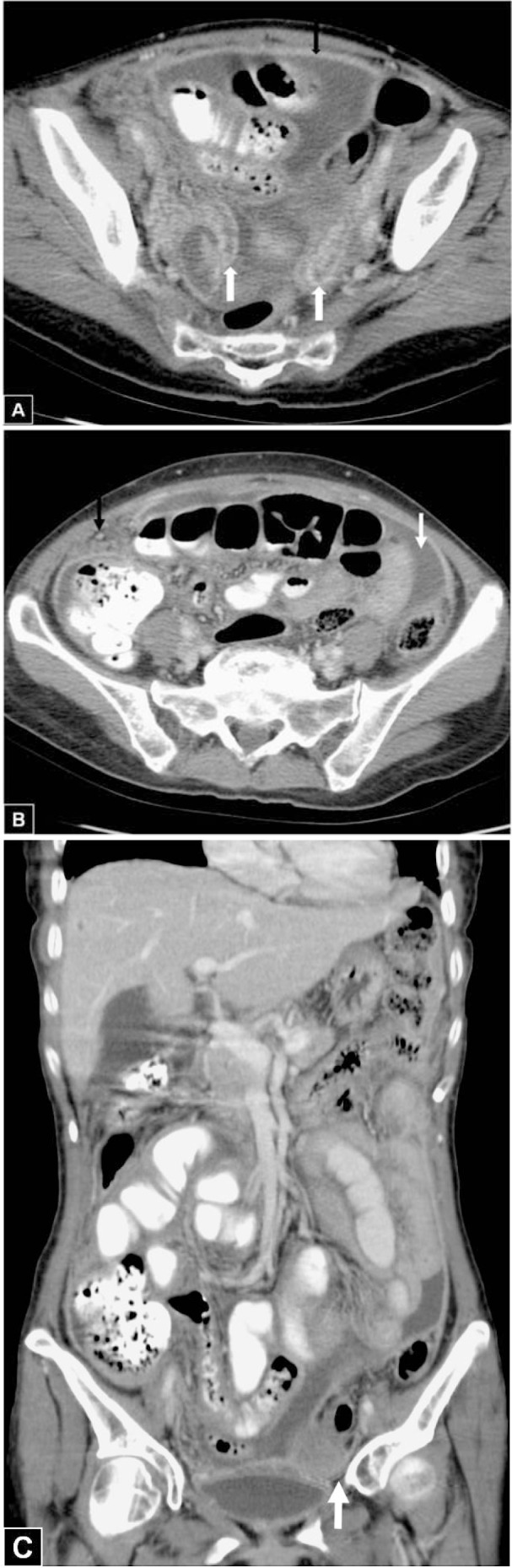
56-year-old woman had suffered from abdominal fullness for three months. (A) Axial contrast-enhanced abdominopelvic CT scan shows a uniform well-enhanced peritoneum (black arrow), and bilateral dilated convoluted fallopian tubes with intense mucosal enhancement (white arrow) representing bilateral salpingitis; these findings combined with dirty fat strandings identify infection. (B) Axial contrast-enhanced CT scan shows the nodules of the omentum (black arrow) and loculated ascites (white arrow). (C) Coronal contrast-enhanced CT scan demonstrates the disproportionate left ovarian mass (arrow) with loculated ascites, in contrast to the usual findings of ovarian cancer.

A 56-year-old woman had suffered from abdominal fullness for three months. She had had poor appetite, general malaise, nausea and vomiting two weeks earlier, but denied tarry stool or diarrhea. She had lost about 6 kg over 3 months. Abdominal sonography revealed ascites. Laboratory data showed normocytic anemia, with hemoglobin 10.3 g/dL (normal range, 12-16 g/dL), and CA-125 level elevated to 188.6 U/mL (normal range, <35 U/mL). Abdominopelvic CT revealed multiloculated ascites in her abdomen and pelvis, thickened peritoneum, strandings in the omentum, small mesenteric nodules, enlarged mesenteric and paraaortic lymph nodes, prominent ovaries, and dilated fallopian tubes (Figure 1(Fig. 1)). There was no evidence of soft tissue in the ileocecal region. Under the initial diagnosis of ovarian cancer with carcinomatosis peritonei, she was admitted and exploratory laparotomy was performed. The operative findings included small nodules in the peritoneum, omentum, small bowel loops, uterus, and fallopian tubes and severe adhesions between bowel loops, the left ovary, and the pelvic side wall. The tentative diagnosis was carcinomatosis peritonei, so left salpingo-oophorectomy, enterolysis and peritoneum biopsy was performed.

**Figure 2:**
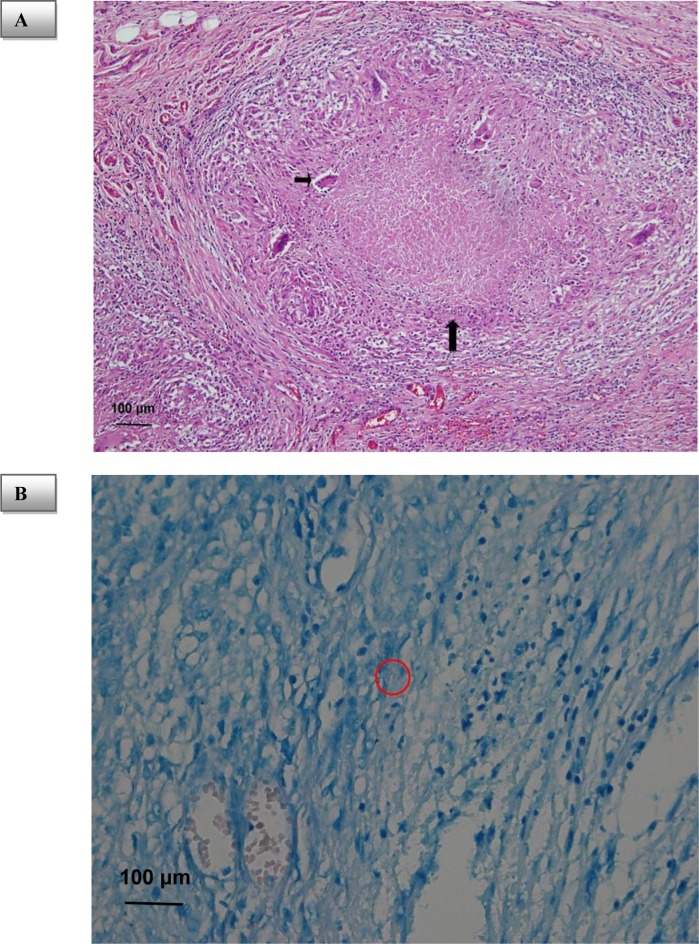
Photograph of histological specimen. (A) Hematoxylin and eosin stain shows the caseous necrosis (long arrow) and Langhans giant cell (short arrow) confirming tuberculosis peritonitis. (B) Acid-fast stain shows the tubercle bacilli (red circle)

The final diagnosis from the biopsy was tuberculosis peritonitis. Microscopic sections showed granulomatous inflammation with central caseous necrosis, epithelioid histiocytes and Langhans giant cells in the ovary, fallopian tube, and peritoneum (Figure 2A(Fig. 2)). Mycobacterial bacilli were also demonstrated by acid-fast stain (Figure 2B(Fig. 2)). 

## Discussion

Tuberculous peritonitis is a rare manifestation of tuberculosis, occurring in less than 4 % of tuberculosis patients (Thoeni and Margulis, 1979[11]). It is still a very important cause of ascites in endemic areas. In industrialized countries, patients with alcoholism, cirrhosis, renal failure, diabetes mellitus or malignancy, or other immunodeficiencies such as AIDS are the high-risk groups (Na-ChiangMai et al., 2008[7]). However, because of the relatively lower incidence of AIDS in female patients and imaging findings similar to those of ovarian cancers, clinicians and radiologists can easily overlook the possibility of tuberculous peritonitis. Tuberculous peritonitis and ovarian cancer are totally different disease entities. With adequate therapy regimens, tuberculous peritonitis has a good prognosis, except in older patients in poor health (Chow et al., 2002[3]). Gynecologists should be alerted to the possibility of tuberculosis infection, and specimens should be taken during operation to avoid unnecessary extended surgery. 

CA-125, the common tumor marker of ovarian cancer, is also elevated in patients with pulmonary and extrapulmonary tuberculosis (Yilmaz et al., 2001[12]). This is because CA-125 antigen is a large transmembrane glycoprotein derived from coelomic (pericardium, pleura, peritoneum) and Mullerian (fallopian tube, endometrial, and endocervical) epithelium. Elevation of CA-125 antigen can be caused by ovarian cancer, other malignancy, benign ovarian disease and benign gynecological conditions, particularly leiomyomas, in female patients (Moss et al., 2005[6]). Bilgin et al. (2001[2]) reported 10 cases of peritoneal tuberculosis with pelvic abdominal masses, ascites and elevated CA-125 mimicking advanced ovarian carcinoma. They concluded that there is a high rate of misdiagnosis between advanced ovarian cancer and peritoneal tuberculosis. 

The most common CT features of tuberculous peritonitis in published reports include high-density lymphadenopathy, high-density ascites, and thickening and nodularity of peritoneal surfaces, mesentery, omentum, and bowel walls. In appearance, there is significant overlapping, and none of these CT findings is pathognomonic for tuberculous peritonitis (Rodriguez and Pombo, 1996[9]; Barutcu et al., 2002[1]). According to the Rodriguez study, the most useful CT findings for distinguishing peritoneal tuberculosis and peritoneal carcinomatosis are in the appearance of the parietal peritoneum (Rodriguez and Pombo, 1996[9]). A smooth peritoneum with minimal thickening and pronounced enhancement suggests peritoneal tuberculosis, whereas nodular implants and irregular peritoneal thickening suggest peritoneal carcinomatosis (Rodriguez and Pombo, 1996[9]). Fallopian tubes are affected in 94 % of women with genital tuberculosis. Salpingitis caused by hematogenous dissemination is almost always bilateral (Leder and Low, 1995[5]). Disproportionate bilateral adnexal masses, dilated and intense enhanced mucosa of bilateral fallopian tubes representing salpingitis, smudged fat planes and loculated ascites are the important clues to a correct diagnosis. CA-125 data are not crucial for ruling out peritoneal tuberculosis because CA-125 can also be markedly elevated by a chronically inflamed peritoneum. 

## Financial interests

None declared. 

## Competing interests

The authors state that they have no Conflict of Interest (COI).
